# Supplementary material for: PATMA: parser of archival tissue microarray
Source: PeerJ. 2016 Dec 1;4:e2741. doi: 10.7717/peerj.2741 (PMC5136132; doi:10.7717/peerj.2741)
Supplement: Supplemental Information 1 — PATMA software source code created and tested in MATLAB R2015b. [file peerj-04-2741-s001.zip › PunchGUI_manual.pdf]

PATMA: Parser of archival TMA

Manual

Ver. 0.2

Authors:

Lukasz Roszkowiak and Carlos Lopez

I. Before you begin

PATMA software was created and tested in MATLAB R2015b. If you are using older version of MATLAB please use caution and be prepared to make adjustments in m-files.

Files:

Please make sure you have all the files in one folder. Currently PATMA consists of 13 files:

1. findEmpties2.m
2. findRowCol2.m
3. getProcOptions.m
4. getRefDistance.m
5. punch_objects.m
6. punch_preprocessing.m
7. punchGUI.fig
8. punchGUI.m
9. punchNumberUpdate.m
10. treatArea2.m
11. treatNotinTable1.m
12. treatOverlap2.m
13. updateTable.m

Add to search path

Please prepare your MATLAB before you begin working with PATMA software. Add folder that contains all the m-files to the MATLAB search path. You can do that either by right-clicking the folder in MATLAB current folder window and selecting “Add to Path -> Selected folder” or by selecting “Set Path” located in the Home toolbar.

How to start

Run ***punchGUI*** in MATLAB command line.

DISCLAIMER:

"I'm not a programmer, I'm just a scientist doing stuff!"

II. Main window

The main window of Graphical User Interface (GUI).

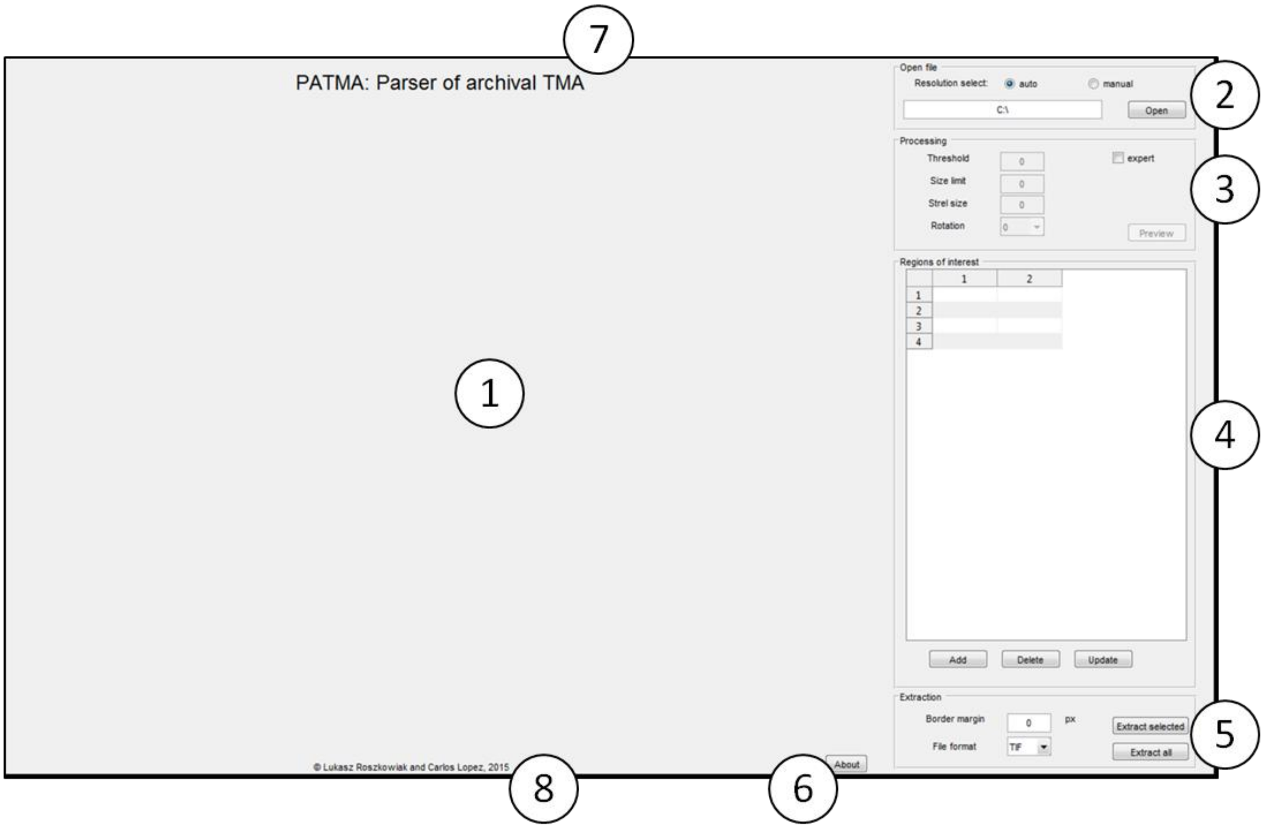

Figure 1 GUI without loaded image.

Element:

#	Element	Description
1	Presentation area	Image of TMA is shown with imposed ROI
2	Open file	Panel
3	Preprocessing	Panel
4	Regions of interest	Panel
5	Extraction	Panel
6	About	Pushbutton – information about the software, project, version and authors
7	Title	Name of the software
8	Authors	Creators of the software

How to use, step by step:

1. Select the method of resolution selection[Auto or Manual].
2. Press [Open] button to select the file.
3. Check expert checkbox.
4. Adjust the [Threshold] value used during binarization.
5. Adjust the [Size limit] of the minimal size of the accepted object.
6. Adjust the [Strel size] of the morphological structuring element.
7. Adjust the [Rotation] to achieve proper orientation.
8. Press [Preview] button to see the binary image where the localization of punches is performed (opened in new Figure).
9. Select all unnecessary ROI (Shift+LeftMouseButton or Ctrl+ LeftMouseButton).
10. Press [Delete] button.
11. Press [Add] button to pick the missing punches.
12. Relocate the new ROI.
13. Repeat steps 3 and 4 for all missing punches.
14. Assign correct validity in the second column of the table.
15. Press [Update] button.
16. Correct punch numbering by editing the first column of the table, if necessary.
17. Press [Update] button.
18. Adjust [Border margin] value used during extraction.
19. Select [File format] for the extracted images.
20. Press [Extract selected] or [Extract all] button to extract images.

Additional comments:

Steps 3-8 are only necessary if the results of finding ROI are very bad. For example if the stain deposit in the background is very high automatic algorithm may calculate too high value of threshold. In that case the values of processing may be adjusted manually.

Steps 9-16 are only necessary if modifications of ROI are applied.

III. Open file

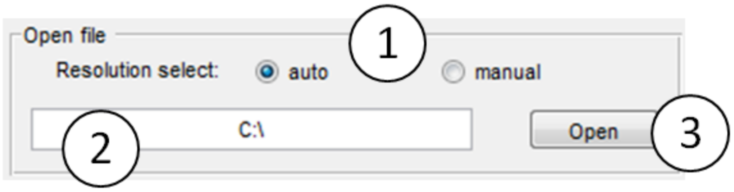

Figure 2 Open file menu

Elements:

#	Element	Type	Description
1	Resolution selection	Radio button	Automatic / manual selection of resolution used for preview and ROI estimation
2	Path line	Display line	Displays path to opened file
3	Open button	Pushbutton	Opens dialog that is used to select the file to open

How to use, step by step:

1. Choose the method of resolution selection. For first try it is better to use Automatic selection.
2. Press [Open] button to select the file.

Additional comments:

Automatic selection of resolution chooses optimal size of image from within pyramidal tiled files. TIF files: select smallest available image. JPEG2000 files: select image with mean of maximum and minimum of Wavelet Decomposition Level.

Manual selection: Displays dialog with all available resolutions to choose from. Typically it is best to use as small resolution as possible to make the calculations faster.

IV. Processing

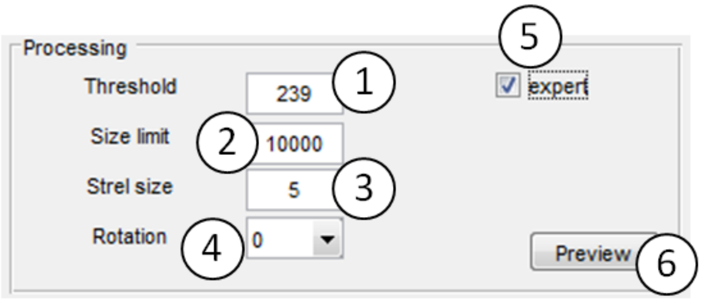

Figure 3 Processing menu

Elements:

#	Element	Type	Units	Description
1	Threshold	Edit field	Intensity level	Threshold used to create binary image where the localization of punches is performed
2	Size limit	Edit field	Pixels	Minimum size of object in binary image that is accepted as punch
3	Strel size	Edit field	Pixels	Radius of the disk-shaped structuring element
4	Rotation	List selection	Angle degrees	Rotate image in a counterclockwise direction around its center point.
5	Expert mode	Checkbox	-	Turn on/off expert mode; enables edit fields in Processing menu
6	Preview	Pushbutton	-	Opens binary image in new Figure

How to use, step by step:

1. Check expert checkbox.
2. Adjust the [Threshold] value used during binarization. Typical values: 230-250.
3. Adjust the [Size limit] of the minimal size of the accepted object. Typical values depend on resolution.
4. Adjust the [Strel size] of the morphological structuring element. Typical values: 2-5.
5. Adjust the [Rotation] to achieve proper orientation. Values: 0, 90, -90, 180.
6. Press [Preview] button to see the binary image where the localization of punches is performed (opened in new Figure).

Additional comments:

Expert mode is switched off by default and values of parameters (1-4) are evaluated automatically. Only when user activates the expert mode with checkbox (5) edit fields in this part are available for editing.

Rotation is performed in counterclockwise direction to perform counterclockwise rotation select -90 degrees. Selected rotation is performed based on the originally loaded image orientation, not on the orientation visible in the GUI.

V. Regions of interest

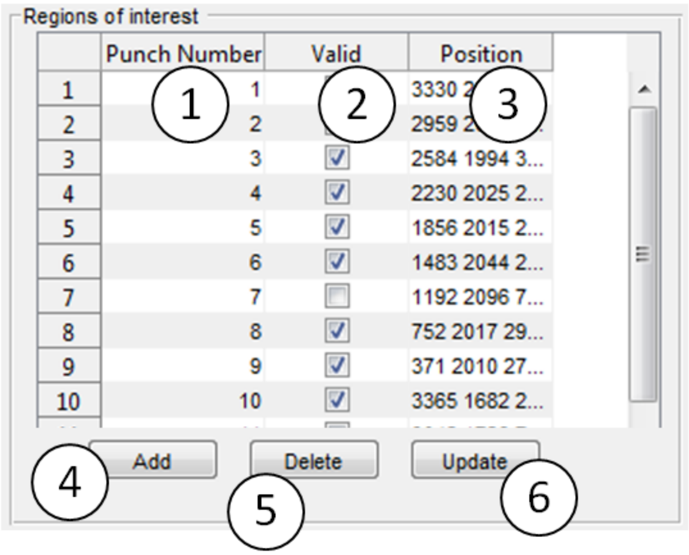

Figure 4 Regions of interest menu (cropped)

Elements:

#	Element	Type	Description
1	Punch Number	Table column	Number assigned to region of interest; edit enabled
2	Valid	Table column	Turn on/off validity of the object; changes visibility of the ROI in the image; objects marked as not valid are enumerated but are not extracted
3	Position	Table column	Size and location of the ROI; position of upper left corner of ROI and its width and height
4	Add	Pushbutton	Adds new ROI to the image and list. New dialog window is opened to select the number of ROI to add.
5	Delete	Pushbutton	Removes selected ROI.
6	Update	Pushbutton	Refresh Position and Punch numbering values of all ROI

How to use, step by step:

1. Select all unnecessary ROI (Shift+LeftMouseButton or Ctrl+ LeftMouseButton).
2. Press [Delete] button.
3. Press [Add] button to pick the missing punches.
4. Relocate the new ROI.
5. Repeat steps 3 and 4 for all missing punches.
6. Assign correct validity in the second column of the table.
7. Press [Update] button.
8. Correct punch numbering by editing the first column of the table, if necessary.
9. Press [Update] button.

Additional comments:

All ROI can be interactively relocated and modified by clicking and dragging in the presented image.

New ROI are located in the upper left corner of the image.

VI. Extraction

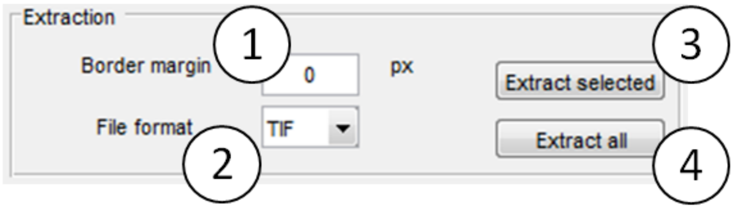

Figure 5 Extraction menu

Elements:

#	Element	Type	Description
1	Border margin	Edit field	Extracted ROI is expanded in every direction by this amount of pixels
2	File format	List selection	Extracted images are saved in this format
3	Extract selected	Pushbutton	Extracts selected ROI
4	Extract all	Pushbutton	Extracts all valid ROI

How to use, step by step:

1. Adjust [Border margin] value used during extraction. Typical value: 1000.
2. Select [File format] for the extracted images.
3. Press [Extract selected] or [Extract all] button to extract images.

Additional comments:

Extracted images are saved in the same directory as the opened filed.

VII. Version update

Ver. 0.1

- Capability of reading TIF pyramidal tiled files.
- Automatic selection of ROI in the image.

Ver. 0.2

- Added JPEG2000 read capability.
- Added extraction file format selection menu.
- Added image rotation option.
- Updated punch numbering method.

Contact

In case of any problems please contact the corresponding author:
Łukasz Roszkowiak (lroszkowiak@ibib.waw.pl)
